# Metagenomic sequencing of CRISPRs as a new marker to aid in personal identification with low-biomass samples

**DOI:** 10.1128/msystems.01038-24

**Published:** 2024-10-29

**Authors:** Kochi Toyomane, Yuri Kimura, Takashi Fukagawa, Takayuki Yamagishi, Ken Watanabe, Tomoko Akutsu, Ai Asahi, Satoshi Kubota, Kazumasa Sekiguchi

**Affiliations:** 1 National Research Institute of Police Science, Kashiwa, Chiba, Japan; University of California, San Diego, La Jolla, California, USA

**Keywords:** CRISPR, human microbiome, personal identification, short tandem repeat

## Abstract

The high specificity of the human skin microbiome is expected to provide a new marker for personal identification. Metagenomic sequencing of clustered regularly interspaced short palindromic repeats (CRISPRs), which we call metaCRISPR typing, was shown to achieve personal identification accurately. However, the intra-individual variability observed in previous studies, which may be due to poor DNA yields from skin samples, has resulted in non-reproducible results. Furthermore, whether metaCRISPR typing can assist in the forensic human DNA analysis of low-biomass samples, from which the information obtained is insufficient, is unknown. In the present study, we sequenced serially diluted control streptococcal CRISPRs cloned into plasmids to determine the minimum copy number required to obtain reproducible results from metaCRISPR typing. We found that at least 10^2^ copies of CRISPRs are necessary to obtain reproducible results. We then analyzed the skin swab samples using both metaCRISPR typing and human DNA typing. When the DNA extracted from the skin swabs was diluted, no information was obtained from six out of eight samples by human DNA typing. On the other hand, beta diversity indices of spacer sequences compared with reference samples were below 0.8 for three out of six samples, for which no information was obtained from human DNA analysis, indicating that the spacers observed in these samples were similar to those in the references. These results indicate that metaCRISPR typing may contribute to the identification of individuals from whom the samples were obtained, even in cases where human DNA yields are insufficient to perform human DNA analysis.

**IMPORTANCE:**

Previous studies have developed new personal identification methods utilizing personal differences in the skin microbiome. However, intra-individual diversity of skin microbiome may preclude the application of microbiome-based personal identification. Moreover, no study has compared microbiome-based personal identification and practical human DNA analysis. Here, we revealed that the results of metaCRISPR typing, a previously developed microbiome-based personal identification method, are stable if the copy number of the marker gene is sufficient. We then analyzed the skin swab samples using both metaCRISPR typing and human DNA analysis. Our results indicate that metaCRISPR typing may provide additional information for personal identification using low-biomass samples that cannot be used for conventional human DNA analysis.

## INTRODUCTION

Currently, forensic personal identification is heavily dependent on short tandem repeat (STR, also called microsatellite) analysis, owing to the high degree of variability (1, 2). In particular, the gold standard for forensic DNA analysis is STR typing using capillary electrophoresis. Although technological advances have improved the sensitivity of STR analysis, STR analysis of low-biomass samples, such as those collected from the surface of objects touched by a person of interest, remains challenging for forensic laboratories (3).

The human skin harbors a commensal bacterial community shaped by the physiological characteristics of the skin (4). According to a recent estimate, the total number of bacterial cells in a man could be in the order of 10^13^, which is comparable to the number of human cells (5). Moreover, in the skin environment, the bacterial DNA load may be greater than the human DNA load because the top layer of the epidermis is composed of enucleated keratinocytes (6). Metagenomic sequencing of skin samples performed by Oh et al. revealed that human-derived sequences accounted for 19.4% ± 6.7% to 98.2% ± 0.1% of the reads, and the majority of DNA was of non-human origin in more than 25% of the palm samples (7). These data suggest that the amount of microbial DNA in the skin, which is mainly composed of bacterial DNA, may be greater than that of human DNA. In addition, bacterial communities on human skin are highly personalized (7, 8) and stable over time (9). Based on this assumption, microbial traces left in the environment can be used to identify individuals who have transferred microbial communities from their skin, even if the samples contain a limited number of human cells (8, 10–18).

Clustered regularly interspaced short palindromic repeats (CRISPRs) are repeat sequences found in prokaryotic genomic DNA that are composed of consensus repeats and unique spacers. CRISPRs are characterized by the integration of invasive DNA, which may have infected prokaryotes in the past as a new spacer, causing the diversity of CRISPR loci among bacterial species. Because of this function, the viral community, which is known to be more personalized than the bacterial community in the human microbiome (19–21), causes the diversity of CRISPR loci among bacterial species, and thus, CRISPRs can be used to differentiate strains of bacterial species (22). Recently, we developed a new microbiome-based forensic individual identification system, in which the spacers of CRISPRs in the skin microbiome were sequenced (18). In the previous study, we identified that CRISPRs of *Streptococcus*, a highly abundant genus in human skin microbiome (4), were also abundant in human skin microbiome. We then showed that amplicon sequencing of streptococcal CRISPRs using primers targeting the repeat sequence, which we call metagenomic CRISPR (metaCRISPR) typing, could more accurately identify a person than the widely used 16S rRNA sequencing.

Although previous studies have revealed the highly personalized nature of spacer diversity in *Streptococcus* CRISPRs, metaCRISPR typing of some skin swab samples derived from the same individuals showed a high Bray–Curtis dissimilarity index, indicating that the diversity of spacer sequences might be non-reproducible (18, 23). Previous studies on 16S rRNA sequencing have revealed the importance of the amount of input DNA for obtaining reliable results from low-biomass samples in microbiome analyses (24–26). Karstens et al. analyzed a dilution series of mock microbial communities and showed that contaminant reads were dominant in the 16S rRNA sequencing data of low-biomass samples (25). In another study, Erb-Downward et al. demonstrated that the 16S rRNA amplicon sequencing data were dominated by stochastic noise if a sample contained <10^4^ copies of the 16S rRNA gene (26). Therefore, we hypothesized that the intra-individual variability observed in metaCRISPR typing was due to the stochastic effect of low biomass. However, whether a minimal number of CRISPR copies is sufficient to obtain reproducible results in metaCRISPR typing is not known. Moreover, no method has been standardized for quantifying CRISPR copies in a sample, such as that for 16S rRNA quantification using qPCR (27–29). Thus, the effect of CRISPR copy number on the results of metaCRISPR typing should be investigated, and the method for quantification of the spacer load in a sample should be developed.

Moreover, few studies have shown that the microbiome in low-biomass samples can be used to identify the person of origin. To our knowledge, in only one study conducted by Schmedes et al., the results of microbiome-based personal identification and human DNA analysis were compared (14), using next-generation sequencing instead of capillary electrophoresis. To utilize microbiome analysis in criminal investigations, proving that microbiome analysis can complement STR analysis is essential for the examination of low-biomass samples, in which the amount of human DNA is limited.

This highlights the need for a qualitative methodology that enables assessment of the quality of microbiome analysis and the importance of demonstrating the advantage of microbiome analysis over human DNA typing for forensic applications. In this study, we assessed the sensitivity of metaCRISPR typing and developed a spacer quantification system to obtain reproducible results. We then examined extremely low-biomass samples using both metaCRISPR typing and conventional human DNA analysis to explore the potential of metaCRISPR typing for personal identification in difficult situations.

## RESULTS

### Assessment of reproducibility of metaCRISPR typing

**Fig 1:**
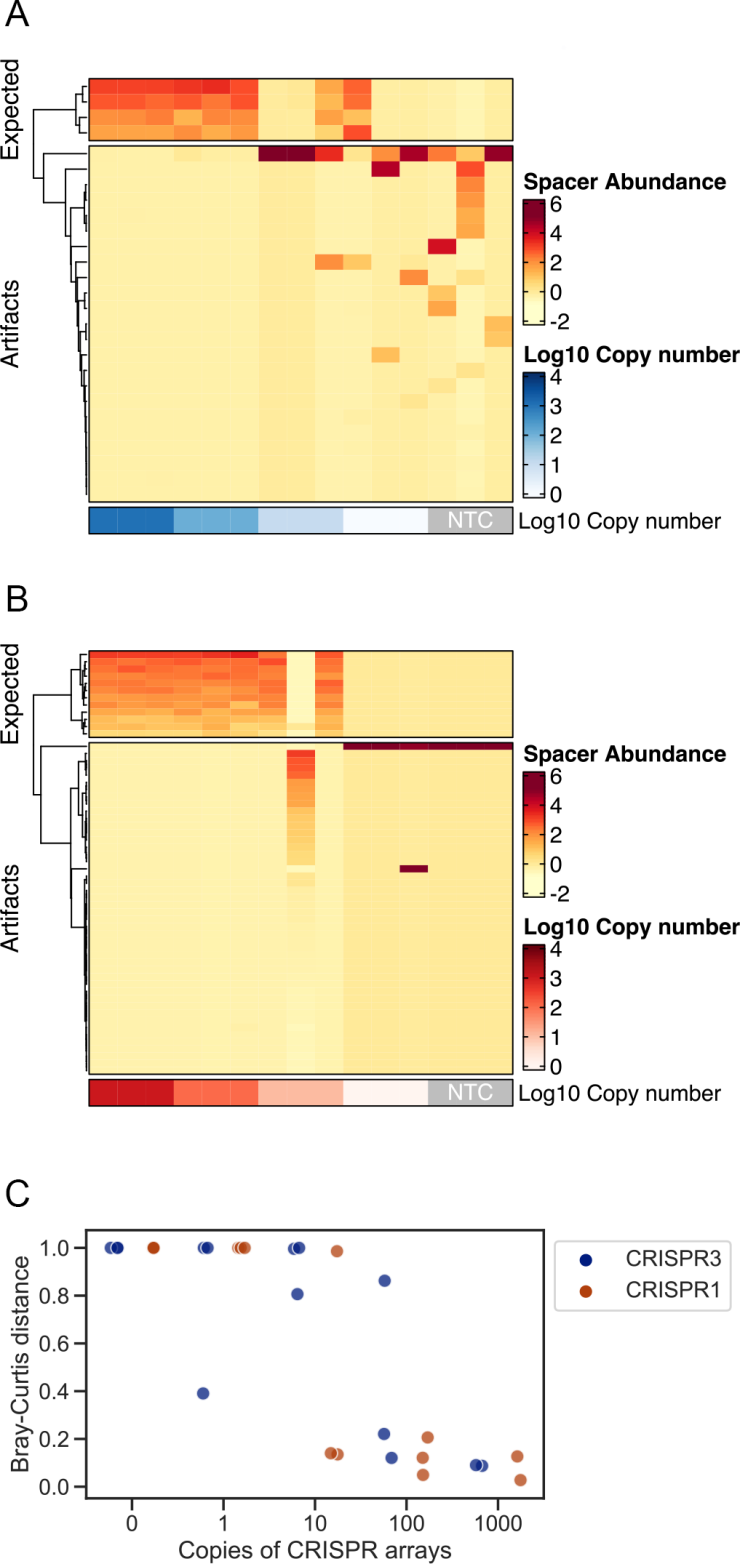
MetaCRISPR typing of serially diluted standard CRISPR arrays. Serially diluted CRISPRs cloned into the pGEM vector were sequenced in triplicates using MiSeq. Heatmap of the observed amplicon sequence variants (ASVs) for (**A**) CRISPR3 and (**B**) CRISPR1 sequencing. The ASVs were grouped by the “ward.D2” method using the ComplexHeatmap package in R (30). The abundance of spacers was standardized as the z-score. (**C**) Relationship between the CRISPR copies in a sample and the reproducibility of the results was measured as the Bray–Curtis distance from a reference sample. One of the samples with the highest copy number was used as a reference for calculating the Bray–Curtis distances.

We hypothesized that the unstable metaCRISPR typing results obtained in the previous study were due to stochastic noise caused by the insufficient number of spacer copies. In 16S rRNA sequencing studies, diluted standards are used to evaluate the sensitivity and reproducibility of the methodology (24–26). However, unlike that for 16S rRNA sequencing, there are no standard CRISPRs to determine the lowest CRISPR copy number that yields reproducible results in metaCRISPR typing. Thus, to evaluate the reproducibility of metaCRISPR typing of *Streptococcus* CRISPRs in the human skin microbiome, we first cloned the entire arrays of CRISPR3 and CRISPR1 from *Streptococcus* as standard CRISPR arrays. We successfully obtained two pGEM vectors, pGEM-CRISPR3cont and pGEM-CRISPR1cont, which contained 4 and 12 spacers of CRISPR3 and CRISPR1, respectively (Text S1). The standard *Streptococcus* CRISPRs were then serially diluted from 10^3^ copies/μL to 10^0^ copies/μL and sequenced in triplicates using a MiSeq instrument. Although we obtained 35,348 reads per sample, the number of reads dropped to 22,103 after read trimming (Table S1). Notably, an average of 17,910 reads were obtained from no template control (NTC) samples by CRISPR3 analysis, suggesting that a high number of noise sequences can be observed in low-biomass samples. As expected, a clear relationship between the CRISPR copy number and the reproducibility of spacer sequences was observed because the numbers of expected sequences decreased and noise sequences increased as the CRISPR copy number decreased (Fig. 1A and B). We then qualitatively assessed the reproducibility of spacer diversity by calculating the Bray–Curtis distance, a representative beta diversity index that indicates the (dis)similarity between two samples. Bray–Curtis distances from one of the samples with the highest copy number were calculated. We observed that at a particular threshold (10^1^–10^2^ copies), the spacer sequence diversity lost reproducibility and became dominated by noise sequences (Fig. 1C); more than two replicates showed Bray–Curtis distances of <0.8 for 10^2^ copies of pGEM-CRISPR3cont and 10^1^ copies of pGEM-CRISPR1cont, which correspond to approximately 10^2^ copies of spacers considering the spacer copies per CRISPR array.

We also investigated the origin of the noise sequences observed in the NTC samples using a BLAST search (Table S2). CRISPR3 sequencing revealed that noisy sequences comprised sequences similar to those in *Streptococcus* or phage genomes, suggesting that these sequences were derived because of contamination. The most frequently observed noise in CRISPR3 sequencing, which was also observed in the standard *Streptococcus* CRISPR data, was the ACATCTCCGAGCCCACGAGAC sequence. The reverse complement of this sequence completely matches the 5′ end of Illumina’s tag sequence on the reverse primer, GTCTCGTGGGCTCGGAGATGTGTATAAGAGACAG (Table 1), suggesting that this noise was derived from primer dimers. Similar to that in CRISPR3 sequencing, the noise sequence observed in CRISPR1 sequencing was ACTCTCAAGATTTAAGTAACT, which is a partial sequence of the forward primer ilF-CRISPR1_F (Table 1), suggesting that this noise was also derived from primer dimers. No other noise was observed in NTC samples during CRISPR1 sequencing.

**TABLE 1:** Primers used in this study

Purpose	Name	Sequence	Reference
Cloning	CRISPR3_F	GAATGGCGCGATTACGAAAT	Original work
	CRISPR3_R	AAATGAGTAGCTTTTAATGT	Original work
	CRISPR1_F	TGCTGAGACAACCTAGTCTCTC	(31)
	CRISPR1_R	GCAACGACAGGAAGCGACCAAA	(31)
Quantification	CRISPR3_DR_F	TCGAAACAACACAGCTCTAAAAC	Original work
	CRISPR3_DR_R	TGTTGTTTCGAATGGTTCCAAAAC	Original work
	CRISPR1_DR_F	CAAGATTTAAGTAACTGTACAAC	Original work
	CRISPR1_DR_R	AAATCTTGAGAGTACAAAAAC	Original work
Sequencing	ilF-CRISPR3_F	TCGTCGGCAGCGTCAGATGTGTATAAGAGACAGTCGAAACAACACAGCTCTAAAAC	(18)
	ilR-CRISPR3_R	GTCTCGTGGGCTCGGAGATGTGTATAAGAGACAGTGTTGTTTCGAATGGTTCCAAAAC	(18)
	ilF-CRISPR1_F	TCGTCGGCAGCGTCAGATGTGTATAAGAGACAGACTCTCAAGATTTAAGTAACTGTACAAC	(18)
	ilR-CRISPR1_R	GTCTCGTGGGCTCGGAGATGTGTATAAGAGACAGCAGTTACTTAAATCTTGAGAGTACAAAAAC	(18)

### Quantification of CRISPR spacers by qPCR

Although we showed that reproducible metaCRISPR typing requires at least 10 copies of CRISPR arrays in a sample, no methodology has been developed to quantify the CRISPR copies. We developed a qPCR method to quantify CRISPR spacer copies to achieve reproducible results for metaCRISPR typing, in which spacers were amplified using primer pairs targeting CRISPR repeats with intercalating dyes. In this system, pGEM vectors containing a single spacer, pGEM-CRISPR3SP6 or pGEM-CRISPR1SP17 (Text S1), were used as standards for the calibration curve (Fig. S1).

Using this system, we quantified spacer abundance in skin swab samples. In addition, we quantified spacer abundance in saliva samples from the same donors, which were considered as high-biomass samples. As shown in Table 2, the mean copy number of the CRISPR3 spacer in skin swab samples was 362.1 copies/μL, whereas that in saliva was 136,955.8 copies/μL. Similarly, the mean copy number of CRISPR1 spacer in skin swab samples was 4,720.9 copies/μL, whereas that in saliva was 454,586.0 copies/μL. As expected, spacer quantification confirmed the high abundance of CRISPRs in the oral environment. Therefore, we used saliva samples as a reference for metaCRISPR typing.

**TABLE 2:** Copy number of spacers for *Streptococcus* CRISPRs and bacterial or human DNA concentrations

Sample ID	Individual		Spacers (copies/μL)		Bacterial DNA (pg/μL)	Human DNA (pg/μL)
		Type	CRISPR3	CRISPR1		
1	P03	Hp-L	510.3	446.2	2.5	4.0
2	P03	Hp-R	1,829.3	1,360.3	4.2	1.9
3	P06	Hp-L	49.6^*a*^	6.5^*a*^	1.4	5.4
4	P06	Hp-R	24.1^*a*^	ND^*b*^	0.4	4.4
5	P01	Hp-L	53.8^*a*^	989.8	55.9	131.3
6	P01	Hp-R	184.8	2,502.5	9.7	38.5
7	P04	Hp-L	1,060.0	5,244.0	7.8	5.3
8	P04	Hp-R	238.1	2,254.7	5.7	4.4
9	P07	Hp-L	118.9	12,827.5	18.4	41.1
10	P07	Hp-R	2,387.8	12,325.7	58.8	934.3
11	P08	Hp-L	7.8^*a*^	1,237.4	5.0	52.5
12	P08	Hp-R	99.5^*a*^	810.2	4.9	38.0
13	P09	Hp-L	27.1^*a*^	2,466.6	8.8	14.6
14	P09	Hp-R	52.5^*a*^	3,079.9	9.6	58.1
15	P10	Hp-L	470.4	386.2	4.9	37.7
16	P10	Hp-R	140.9	495.0	4.9	92.6
17	P11	Hp-L	43.8^*a*^	299.0	1.2	2.7
18	P11	Hp-R	463.1	4,667.0	1.3	4.7
19	P12	Hp-L	25.9^*a*^	298.5	14.9	17.7
20	P12	Hp-R	24.6^*a*^	633.9	6.9	8.8
21	P04	Hp-L	108.9	33,718.9	78.1	201.9
22	P04	Hp-R	44.1^*a*^	17,809.3	35.5	66.7
23	P03	Saliva	105,085.8	178,727.9	1,299.2	4,315.2
24	P06	Saliva	69,332	188,751.7	2,588.2	10,435.4
25	P01	Saliva	349,187	1,341,240.9	23,162.5	16,369.3
26	P04	Saliva	874,576.4	876,025.1	4,792.5	17,199.2
27	P07	Saliva	11,837.3	19,063.5	238.9	815.7
28	P08	Saliva	2,857.1	34,498.1	67.1	364.3
29	P09	Saliva	4,429.6	83,532.9	536.2	2,801.6
33	P10	Saliva	19,854.2	84,035.5	3,995.6	2,641.9
34	P11	Saliva	32,633.3	1,344,976.6	1,769.4	2,682.1
35	P12	Saliva	27,743.4	339,729.3	1,787.0	3,426.4
36	P04	Saliva	8,977.3	509,864.7	2,805.4	1,409.6
37	P09	Object	1,539.6	1,252.7	0.8	28.4
38	P10	Object	2,502.5	5,540.4	0.4	21.8
39	P12	Object	26.7^*a*^	5.3^*a*^	0.3	5.0
40	P04	Object	1,463.1	1,429.6	0.8	28.4

^*a*^ Below the quantification limit.

^*b*^ No amplification.

### Salivary CRISPR diversity is similar to skin CRISPR diversity within an individual

**Fig 2:**
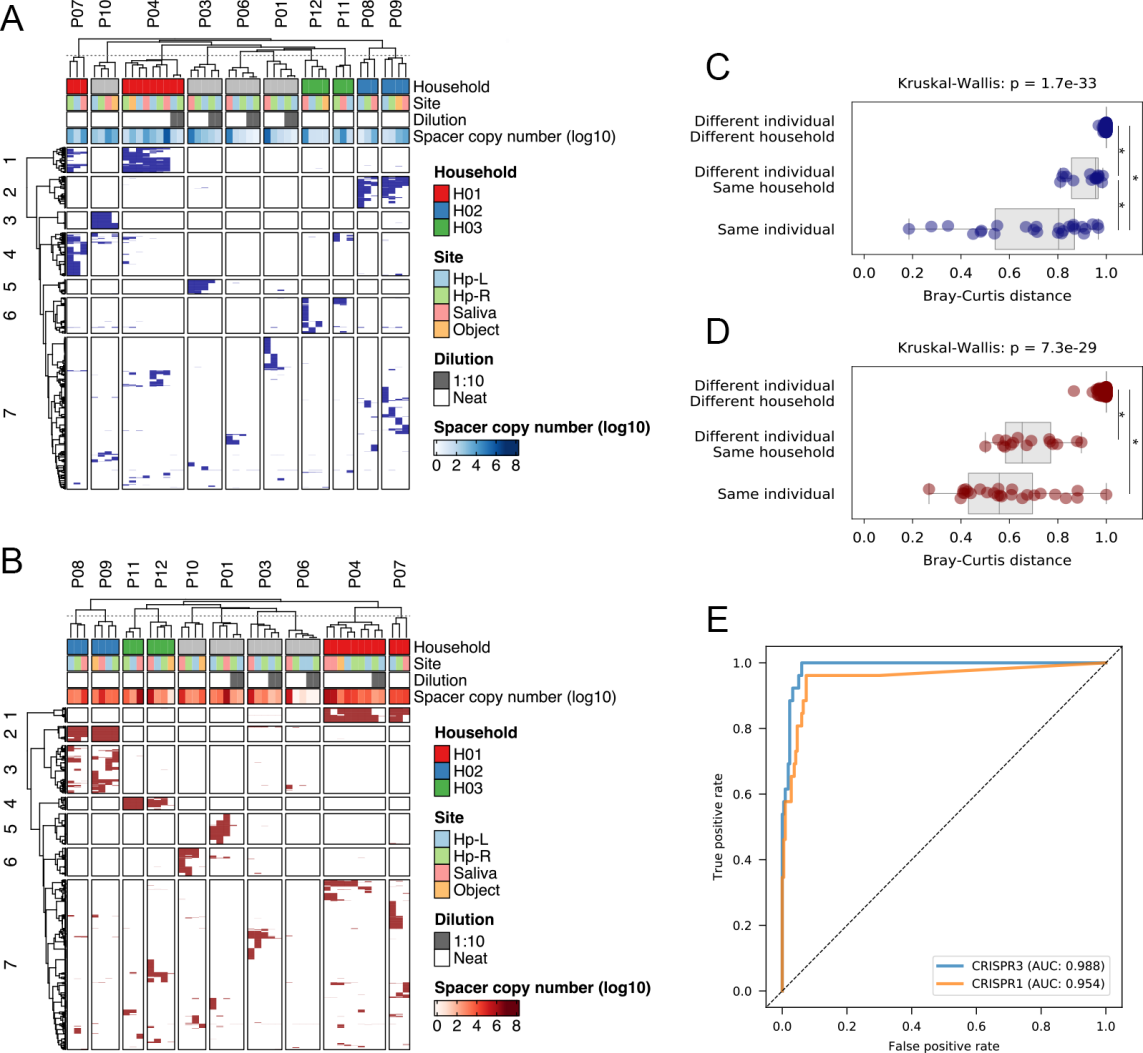
Individual-/household-specific signals obtained by metaCRISPR typing. Skin swabs and saliva samples were analyzed using metaCRISPR typing. The heatmap of the observed ASVs for (**A**) CRISPR3 and (**B**) CRISPR1 sequencing. The columns are grouped according to individuals. The ASVs were grouped by the “ward.D2” method using the ComplexHeatmap package in R (30). The presence (colored)/absence (white) of spacers is indicated. Keyboard swab samples were labeled as “Object.” (**C, D**) Box plots of the Bray–Curtis dissimilarity indices between/within individuals. The indices were compared using the Kruskal–Wallis test and post hoc Mann–Whitney U test with Bonferroni correction (*adjusted *P* < 0.05). (**C**) CRISPR3 and (**D**) CRISPR1. (**E**) ROC curve analysis demonstrating the performance of metaCRISPR typing on personal identification. (**C–E**) Only the Bray–Curtis indices between neat skin swab samples and saliva samples are indicated.

DNA extracted from skin swab samples, along with saliva samples obtained from the same donors as the reference, was subjected to metaCRISPR typing. We also analyzed 10-fold diluted skin swab samples to simulate extremely low-biomass samples. On average, 40,030 reads per sample were obtained from skin and saliva samples (Table S3), and the number of reads for diluted samples was reduced after trimming (a maximum of 98% of the reads were removed). As shown in Fig. 2A and B, specific spacers were observed in both saliva and skin samples obtained from the same individual or different individuals within the same household. This individual-/household-specific similarity in spacer diversity was also confirmed using principal coordinate analysis (Fig. S2). Although obtaining spacer reads from diluted skin swab samples was difficult, specific spacers were also observed in some diluted samples, such as the spacers of CRISPR3 in individual P03. Interestingly, such individual-/household-specific signatures of *Streptococcus* CRISPRs were conserved during 2 years in P04, who provided samples twice in this study after a 2-year interval.

To quantitatively assess the similarity of spacer sequences, the Bray–Curtis distance between skin samples and saliva samples was calculated (Fig. S3). The Bray–Curtis distance between the samples derived from different individuals from different households was consistently high, whereas the value was low for samples derived from individuals within the same household, as shown in Fig. 2C and D. To determine the sensitivity and specificity of metaCRISPR typing for individual identification, receiver operating characteristic (ROC) curve analysis was performed (Fig. 2E). When we set the threshold at 0.967 and 0.882 for CRISPR3 and CRISPR1, we recorded the best sensitivity and specificity (1.0 and 0.940 for CRISPR3, and 0.962 and 0.926 for CRISPR1) with a high area under the ROC curve (AUC) values of 0.988 and 0.954, respectively. While the sensitivity was sufficiently high, we could omit the within-household samples in CRISPR3 and the between-household samples in CRISPR1 which were falsely identified as those belonging to the same individual when the threshold was set at 0.8. Thus, to minimize the false positives, which are the most serious type of error in forensic analysis, we set the tentative threshold at 0.8.

**Fig 3:**
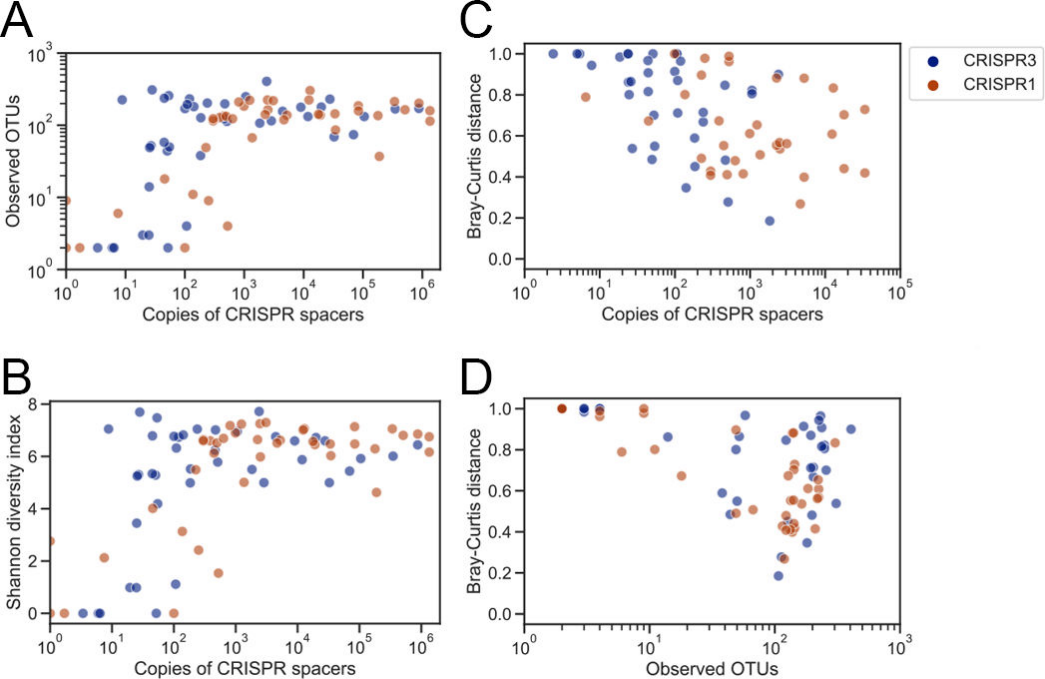
Copy number of spacers as the indicator of quality control for metaCRISPR typing. (**A and B**) Spacer copy numbers in a sample plotted against alpha diversity (A: observed OTUs; B: Shannon diversity index). (**C**) Copy number of spacers in a sample or (**D**) observed OTUs plotted against the Bray–Curtis distance between swab samples and reference saliva samples. (A–D) Data points for neat and 1:10 diluted samples are shown. For visualization, a pseudo-count of one was added if the spacer copy number was not determined.

We also found a strong correlation between the diversity indices, including both alpha and beta diversity indices, and the copy number of spacers in a sample. The alpha diversity indices of spacers, measured as observed operational taxonomic units (OTUs) and the Shannon diversity index, were positively correlated with spacer load (Spearman’s rank correlation coefficient [*r*] of observed OTUs: 0.416 for CRISPR3 and 0.602 for CRISPR1; *r* of Shannon index: 0.359 for CRISPR3 and 0.495 for CRISPR1), indicating that the richness and evenness of spacer diversity were high in spacer-rich samples (Fig. 3A and B). The spacer diversity structure was analyzed by calculating within-individual Bray–Curtis diversity indices. The Bray–Curtis distances between skin swabs and saliva samples derived from the same individuals were calculated. As shown in Fig. 3C, the within-individual Bray–Curtis index was negatively correlated with spacer load in the skin sample (*r*: −0.578 for CRISPR3 and −0.412 for CRISPR1). In contrast to that for spacer loads, the correlation between bacterial DNA load (Table 2) and within-individual Bray–Curtis distance was poor (*r*: −0.387 for CRISPR3 and −0.385 for CRISPR1), as shown in Fig. S4A. These findings suggest that spacer copies of streptococcal CRISPRs, rather than those of total bacterial DNA, were good predictors of the reproducibility of metaCRISPR typing results. Although finding the threshold at which the spacer lost similarity to the reference was difficult, at least 10 copies/μL of spacers in a sample were required to obtain informative results in terms of beta diversity (Fig. 3A), and these results were comparable to those of high-biomass samples at 100 copies/μL in terms of alpha diversity (Fig. 3A and B). In addition, the similarity of the metaCRISPR typing results was also correlated with the number of observed OTUs (*r*: −0.479 for CRISPR3 and −0.454 for CRISPR1), as shown in Fig. 3D. Thus, the spacer diversity loses its structure if the copy number of CRISPRs is insufficient, as expected from the reproducibility assessment performed using pGEM-CRISPR3cont and pGEM-CRISPR1cont.

### Potential complementation of human DNA profiling by metaCRISPR typing

Given the high abundance of *Streptococcus* in the human skin microbiome (4), metaCRISPR typing of *Streptococcus* CRISPRs may complement partial human DNA profiling, resulting in inconclusive results in criminal investigations. Based on this assumption, metaCRISPR typing may be used to obtain information on personal identity using samples with extremely low-biomass DNA, from which no information can be obtained by STR typing. To validate this hypothesis, DNA extracted for metaCRISPR typing was subjected to human DNA profiling using GlobalFiler kit, in which 21 highly polymorphic autosomal STR loci were typed using capillary electrophoresis. To simulate extremely low-biomass DNA samples, 10-fold diluted samples were also tested.

As shown in Table 3, full profiles were obtained for only one sample among eight neat skin swab samples collected from four participants, whereas the overall rate of correctly called loci was high among neat samples (mean: 83%). Incorrect loci comprised loci showing both loss of correct peaks and addition of extra peaks, which include well-known artifacts in STR typing, such as stutter peaks (32). Partial electropherograms of representative samples are shown in Fig. S5. The worst result was observed for sample 2, which was collected from the right hand of individual P03 (57%), reflecting the lowest degree of human DNA recovery (Table 2). Notably, metaCRISPR typing of sample 2 revealed low intra-individual Bray–Curtis distances (0.19 for CRISPR3 and 0.51 for CRISPR1), indicating that the spacer diversity of sample 2 was similar to that of the saliva sample obtained from the same individual. STR typing results of the diluted samples were worse than those of the neat samples (Table 4). Almost no peaks were obtained, except those obtained from sample 5, which had the highest human DNA load. However, personal signatures can be obtained by the meta-CRISPR typing of several diluted samples, as mentioned above. Specifically, CRISPR3 analysis of sample 2 and CRISPR1 analysis of samples 1 and 8 revealed low Bray–Curtis distances, even if the samples were diluted. The correlation between human DNA amounts (Table 2) and within-individual Bray–Curtis distances was poor (*r*: −0.368 for CRISPR3 and −0.360 for CRISPR1), as shown in Fig. S4B, suggesting that the CRISPR profile may be obtained from samples with insufficient human DNA. Notably, while the sensitivity of metaCRISPR typing decreased to 0.2 for CRISPR3 and 0.7 for CRISPR1, the specificity values of the test were 1.0 and 0.936, suggesting that the specificity of the test are not affected by dilution (Fig. S6).

**TABLE 3:** Comparison of metaCRISPR typing and STR typing using neat skin swab samples^*a*^

Sample ID	Individual	Site	metaCRISPR typing result (Bray–Curtis distance)		STR typing results		
			CRISPR3	CRISPR1	Correct loci	Correct loci rate (%)
1	P03	Hp-L	**0.28**	**0.55**	21	100
2	P03	Hp-R	**0.19**	**0.51**	12	57
3	P06	Hp-L	**0.48**	**0.79**	20	95
4	P06	Hp-R	0.86	1.00	17	81
5	P01	Hp-L	**0.55**	**0.61**	20	95
6	P01	Hp-R	**0.45**	**0.54**	13	62
7	P04	Hp-L	0.82	**0.40**	19	90
8	P04	Hp-R	**0.67**	**0.55**	18	86

^*a*^ Values in bold indicate that the metaCRISPR typing results were similar (Bray–Curtis distance < 0.8) to those of the reference saliva sample.

**TABLE 4:** Comparison of metaCRISPR typing and STR typing using 1:10 diluted skin swab samples^*a*^

Sample ID	Individual	Site	metaCRISPR typing result (Bray–Curtis distance)		STR typing results	
			CRISPR3	CRISPR1	Correct loci	Correct loci rate (%)
1	P03	Hp-L	1.00	**0.67**	0	0
2	P03	Hp-R	**0.59**	0.80	0	0
3	P06	Hp-L	1.00	1.00	0	0
4	P06	Hp-R	1.00	1.00	0	0
5	P01	Hp-L	1.00	1.00	19	90
6	P01	Hp-R	0.98	0.98	3	14
7	P04	Hp-L	1.00	0.96	0	0
8	P04	Hp-R	1.00	**0.49**	0	0

^*a*^ Values in bold indicate that the metaCRISPR typing results were similar (Bray–Curtis distance < 0.8) to those of the reference saliva sample.

To test whether metaCRISPR typing can be used to identify the personal signals from the surface of an object touched by the person of interest, we performed metaCRISPR typing on swabs of keyboards owned by representative participants. As expected, person-/household-specific signals, such as the spacers cluster 1 of CRISPR3 in individual P04, were also observed in keyboard-swab samples (Fig. 2A and B). The exceptions were those obtained from P12, whose samples showed the lowest spacer copies. The specificity of the metaCRISPR typing was also confirmed by comparing Bray–Curtis indices between keyboard-swab and reference saliva (Fig. S7). Thus, these results validate our hypothesis that STR typing can be complemented with metaCRISPR typing for extremely low-biomass samples.

## DISCUSSION

In 2010, Firer et al. proposed the use of the microbiome to link touched objects to specific individuals based on the assumption that recovery of bacterial DNA from the surfaces of touched objects would be easier than that of human DNA (8). Other reports have also confirmed the potential of skin microbiome contents as personal identification markers (10–18). However, studies comparing microbiome-based forensic identification and conventional human DNA typing, such as that by Schmedes et al. (14), are limited. Thus, whether microbiome-based personal identification can be used to link touched objects with the individuals who touched these objects, in cases where human DNA typing fails because of the extremely limited amount of human DNA, is unknown. In this study, skin swab samples were analyzed using both the previously developed metaCRISPR and human DNA typing methods, revealing that metaCRISPR typing may help in identifying a person of interest using a low-biomass sample, from which no information was obtained by human DNA typing (Table 4). Although no diluted sample showed a Bray–Curtis index score below 0.8 in both CRISPR3 and CRISPR1 analyses, a Bray–Curtis index score below 0.8 in one streptococcal CRISPR analysis may sufficiently discriminate the person of interest, considering the high level of individual specificity observed in a previous study (18) and this study (Fig. 2C and D). To our knowledge, this is the first study to compare microbiome-based forensic identification with conventional capillary electrophoresis-based human DNA typing.

We also developed a quantification system for spacer copies to evaluate the results of metaCRISPR typing. For 16S rRNA sequencing, the importance of input DNA load is well established. For example, Erb-Downward showed a clear relationship between 16S rRNA copy number and reproducibility of the results of 16S rRNA sequencing and revealed that 10^4^ copies of 16S rRNA are required to obtain reproducible results, in which sequencing noise is limited (26). In this study, we also examined the relationship between CRISPR loads and the reproducibility of the results of metaCRISPR typing using control CRISPR arrays to determine the minimal copy number of spacers required to obtain reproducible results. The sequencing results became dominated by noisy sequences as the copy number of CRISPRs decreased, revealing that at least 10^1^ copies of CRISPR arrays or 10^2^ copies of spacers were necessary for reproducible metaCRISPR typing. Similar to that for the control CRISPR arrays, the alpha diversity indices decreased, and within-individual Bray–Curtis distances increased when the spacer copy number was below 10^1^ copies in skin swab samples, indicating that insufficient copies of CRISPRs decrease the reproducibility of metaCRISPR typing. The results of keyboard swab samples also supported a minimal concentration of spacer load because a limited number of individual-specific spacers were observed in the samples of P12, which showed the lowest spacer loads among keyboard samples (Fig. 2A and B; Table 2). These data suggest that qualitative assessment of spacer copies prior to metaCRISPR typing is important for achieving a reproducible analysis. To this end, we propose 10^2^ copies as the minimal copy number of spacers required to obtain reliable data for metaCRISPR typing, and metaCRISPR typing may provide informative results when insufficient human DNA is recovered.

In this study, the origins of noisy sequences were identified using a BLAST search. The noisy sequences observed in the NTC samples included *Streptococcus* and streptococcal phage genomes, suggesting a baseline level of contamination. Low-level contamination by environmental DNA has also been reported in other skin microbiome-based personal identification systems (14). However, the most frequently observed noise was derived from primer dimers. Although we performed the purification of the constructed libraries to reduce the number of primer dimers, elimination of all dimers was difficult because the insert size was smaller than that in typical amplicon sequencing (typical insert sizes were 66 bp for CRISPR3 and 88 bp for CRISPR1, whereas those in 16S rRNA sequencing are >300 bp). Thus, these data suggest the importance of quality control, such as the quantification of spacer copies, in metaCRISPR typing.

We propose the use of saliva samples as references for meta-CRISPR typing. The oral microbiome is rich in CRISPRs compared to that in other body sites, such as the gut, skin, and urogenital body areas. Furthermore, streptococcal CRISPR-associated 1 (Cas1), which integrates new spacers into the CRISPR-Cas system, is abundant in the oral environment (33). Moreover, spacers are more conserved in saliva than in skin and shared between the saliva and skin (34). Thus, saliva samples were used as candidate reference samples, in which the copy number of the CRISPR spacer was expected to be high. As expected from previous studies, the spacer sequences of streptococcal CRISPRs in saliva samples were rich in copy numbers (Table 2) and highly similar to those in skin swab samples (Fig. 2A and B), supporting the use of saliva as a reference for metaCRISPR typing. As proposed above, low Bray–Curtis distances between skin swabs and reference saliva samples, such as values below 0.8, may be used as a signal for personal identity. Notably, our results indicated that spacer sequences were shared between household members (Fig. 2A through D), which is consistent with the previous reports demonstrating the sharing of spacers among household members (35, 36). While sequences of *Streptococcus* CRISPR1 were similar among cohabiting couples, within-household Bray–Curtis distances of *Streptococcus* CRISPR3 were higher than those within individuals (Fig. 2C and D). Our results suggested that qualitative assessment of *Streptococcus* CRISPR3 can be used to identify person-specific markers. However, the sharing of spacers among household members may limit the application of metaCRISPR typing on discrimination of household members.

An important aspect of the microbiome, in contrast to human DNA contents, is that it can change over time. While the skin microbiome community is specific to the individual and remains stable over time (9), it is not clear if the personal-specific signature of the skin microbiome persists after a long period. Longitudinal analysis was not performed in this study. However, our previous study showed that the same spacers were observed in the same individual after a 6-month interval, suggesting longitudinal stability of *Streptococcus* CRISPRs (23). This stability of CRISPR spacers is also in line with the temporal stability of the phage community observed over 2 months (21). The stability of the spacer over 2 years in P04 in this study also supports the longitudinal stability of CRISPRs. This short-term stability may be sufficient for most case investigations, except for cold cases.

One of the most challenging aspects of metaCRISPR typing is that the potential error rate of metaCRISPR typing is not clear, which is required to evaluate the reliability of a scientific technique to be used for forensic purposes (37). Although we determined the potential sensitivity and specificity of the methodology, these values apply to the limited population studies. For forensic techniques, such as DNA analysis or latent fingerprint examination, large-scale evaluations have been performed to assess the accuracy of the techniques (38–40). Such large-scale validation should be performed in future studies to assess the discriminatory power of metaCRISPR typing. In addition, we have previously found that other skin microbiome members, such as *Neisseria* and *Veillonella*, also harbor CRISPRs in their genome (18). Since the percentage of shared spacers among household members may be different between CRISPR systems (35), exploring other CRISPRs or a combination of more CRISPRs may improve our approach to discrimination of household members. Although further studies are required, metaCRISPR typing may be used as a new tool to assist human DNA analysis in cases where little or no information is obtained from the samples, which may contribute to criminal investigations. Recent studies have highlighted the potential use of CRISPRs for strain-level tracking (36, 41). Our results also support the use of CRISPRs for strain-level bacterial community analysis, which may contribute to the understanding of the complex structure of bacterial communities influenced by social interactions.

## MATERIALS AND METHODS

### Sample collection

Skin swabs and saliva samples were obtained from Japanese volunteers noninvasively. In this study, samples were collected in two phases: Phase 1 in September 2022 and Phase 2 in June 2024. One participant (P04) provided samples in both phases. In phase 2, samples were collected from three pairs of cohabiting couples, namely, P04 and P07, P08 and P09, and P11 and P12, who shared households.

Skin swab samples were collected from approximately 5 × 5 cm regions of the left and right hypothenar palms (Hp) of 10 individuals (*n* = 22) using cotton-tipped swabs moistened with water, as previously described (18). In the Phase 2 sample collection, keyboard samples (*n* = 4; the samples were collected from the surface of the space key on the individual’s keyboard) were also collected using cotton-tipped swabs moistened with water. Saliva samples (*n* = 11) were collected in sterile 50 mL tubes from the same volunteers. All the samples were stored at −80°C before the DNA extraction, which was performed using the EZ1 DNA Investigator kit (EZ1; Qiagen, Hilden, Germany) or DNeasy PowerSoil Kit (PS; Qiagen) according to the manufacturer’s instructions. From swab samples, DNA was extracted using EZ1 or PS as described previously (23). For the saliva samples, 50 µL of saliva was used as input. DNA was subsequently extracted using EZ1 or PS with an elution volume of 50 µL.

Bacterial DNA was quantified from all skin swabs/saliva samples using a Femto Bacterial DNA Quantification Kit (Zymo Research, Irvine, CA, USA) with QuantStudio 5 (Thermo Fisher Scientific, Waltham, MA, USA) in accordance with the manufacturer’s instructions.

### Cloning of CRISPRs

CRISPR arrays cloned into the pGEM-T-easy vector (Promega, Madison, WI, USA) were used to evaluate the sensitivity of metaCRISPR typing. Primer pairs used to amplify the CRISPR arrays are listed in Table 1. To quantify the CRISPR arrays, we also cloned spacers of the CRISPRs using the sequencing primers listed in Table 1 into the pGEM-T-easy vector. The sequences of the inserts were verified by Sanger sequencing and are provided in Text S1 of the supplemental material. The concentration of the resulting plasmids was determined using a NanoDrop One instrument (Thermo Fisher Scientific).

### Quantification of CRISPR spacers

To quantify the two *Streptococcus* CRISPRs, spacer quantification was performed by qPCR using TB Green Premix Ex Taq (Tli RNaseH Plus; Takara Bio, Shiga, Japan) and QuantStudio 5 with primer pairs targeting repeat sequences of the CRISPRs (Table 1). The standard curve was generated by serially diluting spacer-containing plasmids (pGEM-CRISPR3SP6 for CRISPR3 and pGEM-CRISPR1SP17 for CRISPR1; Text S1) from 10^6^ copies/μL to 10^2^ copies/μL. Each reaction was performed in a reaction volume of 10 µL containing 5 µL of 2× TB Green Premix Ex Taq (Tli RNaseH Plus), 0.2 µM of each primer, 1× ROX II dye, and 1 µL of genomic DNA. An NTC was used for each reaction. The following conditions were used for amplification: 30 s at 95°C, 40 cycles of denaturation at 95°C for 5 s, annealing and extension at 64°C (for CRISPR 3) or 60°C (for CRISPR1) for 34 s, followed by melting curve analysis from 60°C to 95°C. Each qPCR was performed in duplicates.

### MetaCRISPR typing

MetaCRISPR typing was performed as previously described (18). In this study, two CRISPRs of *Streptococcus* were analyzed. The spacer regions of the two streptococcal CRISPRs were amplified using the primer pairs listed in Table 1. For dilution experiments, plasmids containing an entire CRISPR array (pGEM-CRISPR3cont for CRISPR3 and pGEM-CRISPR1cont for CRISPR1) were serially diluted from 10^3^ to 10^0^ copies/μL. Then, 1 µL of plasmids or PCR-grade water (NTC) was used as a template. PCR amplification for the dilution experiments was performed in triplicates. For skin/saliva sample analysis, 4 µL of extracted DNA was used as a template. Amplicons were purified using the Agencourt AMPure XP kit (Beckman Coulter, Brea, CA, USA) and indexed using the Nextera XT DNA index kit (Illumina, San Diego, CA, USA). The libraries were diluted to 4 nM, based on the concentration determined using a GenNext NGS Library Quantification Kit (Toyobo, Osaka, Japan). If the library concentration was less than 4 nM, the library was pooled with other libraries without dilution. Paired-end sequencing was performed for 300 cycles (2 × 151) on a MiSeq platform (Illumina) using MiSeq v2 chemistry according to the manufacturer’s instructions. The sequence data obtained in this study have been deposited in the Sequence Read Archive under the accession number PRJNA1070389.

### Human DNA typing

Human DNA was quantified using a QuantiFiler HP DNA Quantification Kit (Thermo Fisher Scientific) with QuantStudio 5, according to the manufacturer’s instructions. STR typing was performed using the GlobalFiler PCR Amplification Kit (Thermo Fisher Scientific) according to the manufacturer’s instructions, with a 29-cycle protocol. The template DNA was 15 µL unless the total amount of input DNA exceeded 1 ng. If the total DNA content was >1 ng, 1 ng of template DNA was used. Subsequently, 1 µL of the PCR amplicon was separated by electrophoresis on a 3500xL Genetic Analyzer (Thermo Fisher Scientific) with 9.6 µL of Hi-Di formamide and 0.4 µL of 600 LIZ dye size standard v2.0.

### Data analysis

Data analyses for meta-CRISPR typing were performed as described previously with some modifications (18). Initially, low-quality reads (Q < 20) and primer sequences were removed and reads shorter than 20 bp or longer than 40 bp were filtered using the CLC Genomics Workbench 23.0.2 (Qiagen). The remaining spacer sequences were analyzed using QIIME2 v2020.2 (42). Spacer reads were denoised and dereplicated using the denoise-paired method of the q2-dada2 plug-in (43), with min_fold_parent_over_abundance = 32. The resulting amplicon sequence variant (ASV) table was further analyzed. The alpha-/beta-diversity index was calculated using the q2-diversity plug-in. To determine the sensitivity and specificity of metaCRISPR typing, ROC curve analysis was performed using the Python library “scikit-learn” (version 0.22.1). Two samples were regarded as similar if the Bray–Curtis dissimilarity between them was below 0.8, a tentative threshold defined in this study, based on the distribution of Bray–Curtis dissimilarity indices of spacer diversity in a previous report (18) and the ROC curve analysis. For visualization, the ASV table was standardized by calculating the z-score or binarized and incorporated into R (version 4.0.2). A heatmap was generated from the table using the ComplexHeatmap library (version 2.6.2) to visualize spacer diversity (30). To determine the origin of noise sequences, a BLAST search was performed using Biopython (version 1.77) (44, 45). The hit with the lowest e-value was recorded.

For human DNA typing, data were analyzed using GeneMapper ID-X software v1.4 (Thermo Fisher Scientific). In this study, the threshold value was set at 175 RFU. We analyzed 21 autosomal loci among 24 loci amplified using the GlobalFiler kit.

All statistical tests were performed using the “stats” module of the Python library “SciPy” (version 1.3.1). To compare the Bray–Curtis distance among individuals, Kruskal–Wallis tests were performed. Then, post hoc pairwise comparisons were performed by the Mann–Whitney U test with Bonferroni correction. For correlation analysis, Spearman’s rank correlation coefficients were calculated. Spearman’s rank correlation coefficients were indicated only when *P*-values were smaller than 0.05.

## SUPPLEMENTAL MATERIAL

Text S1 msystems.01038-24-s0001.txt FASTA-formatted sequences of the plasmids used in this study.

Supplemental Figures msystems.01038-24-s0002.pdf Fig. S1 to S7; caption for Text S1.

Supplemental Tables msystems.01038-24-s0003.xlsx Tables S1 to S3.

ASM does not own the copyrights to Supplemental Material that may be linked to, or accessed through, an article. The authors have granted ASM a non-exclusive, world-wide license to publish the Supplemental Material files. Please contact the corresponding author directly for reuse.
