# Supplementary material for: Metagenomic sequencing of CRISPRs as a new marker to aid in personal identification with low-biomass samples
Source: mSystems. 2024 Oct 29;9(11):e01038-24. doi: 10.1128/msystems.01038-24 (PMC11575304; doi:10.1128/msystems.01038-24)
Supplement: Supplemental Figures — Fig. S1 to S7; caption for Text S1. [file msystems.01038-24-s0002.pdf]

Supplemental Material
Text S1
FASTA-formatted sequences of the plasmids used in this study. Only the sequences between the T7 and SP6 sites are shown.

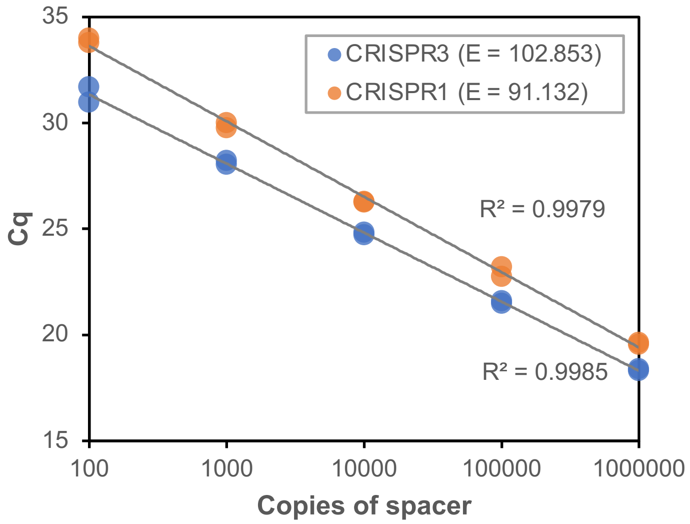

Supplemental Figure S1

Calibration curves for spacer qPCR. “E” denotes PCR efficiency.

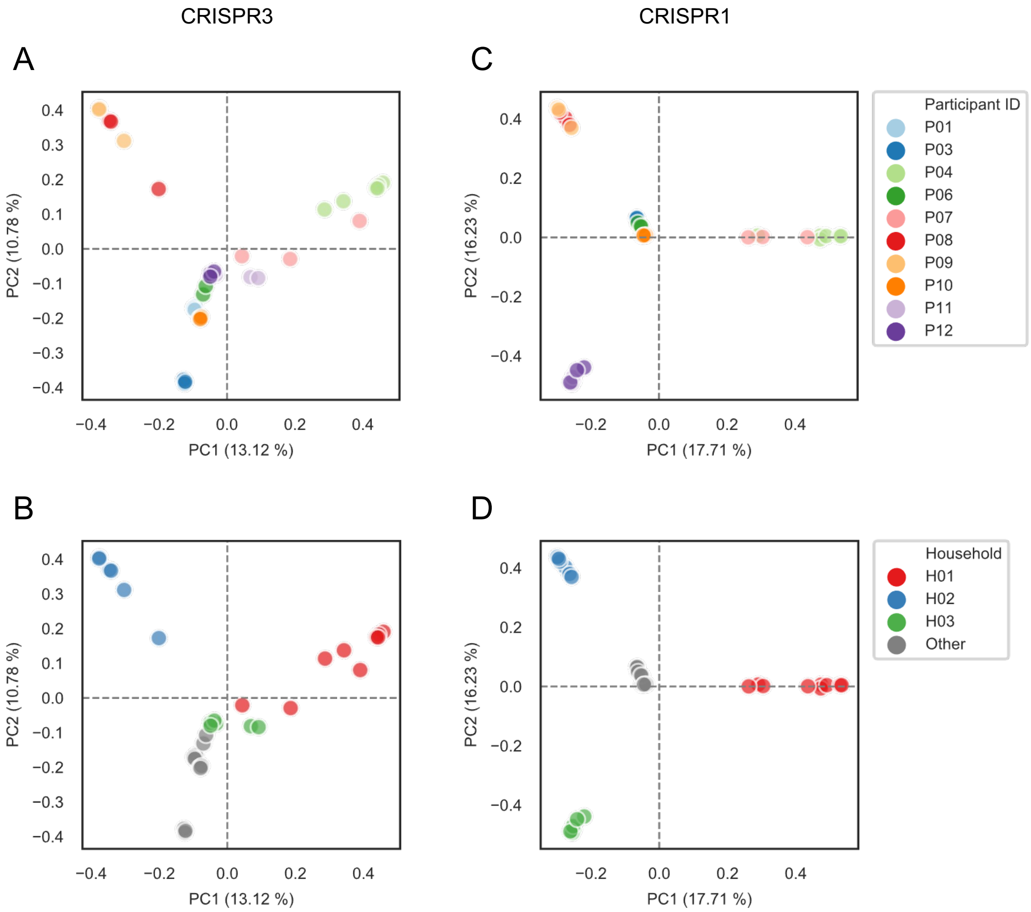

Supplemental Figure S2

Principal coordinate analysis of the spacers. (A and B) CRISPR3 and (C and D) CRISPR1 sequences. Each data point is colored according to (A and C) the individual or (B and D) the household.

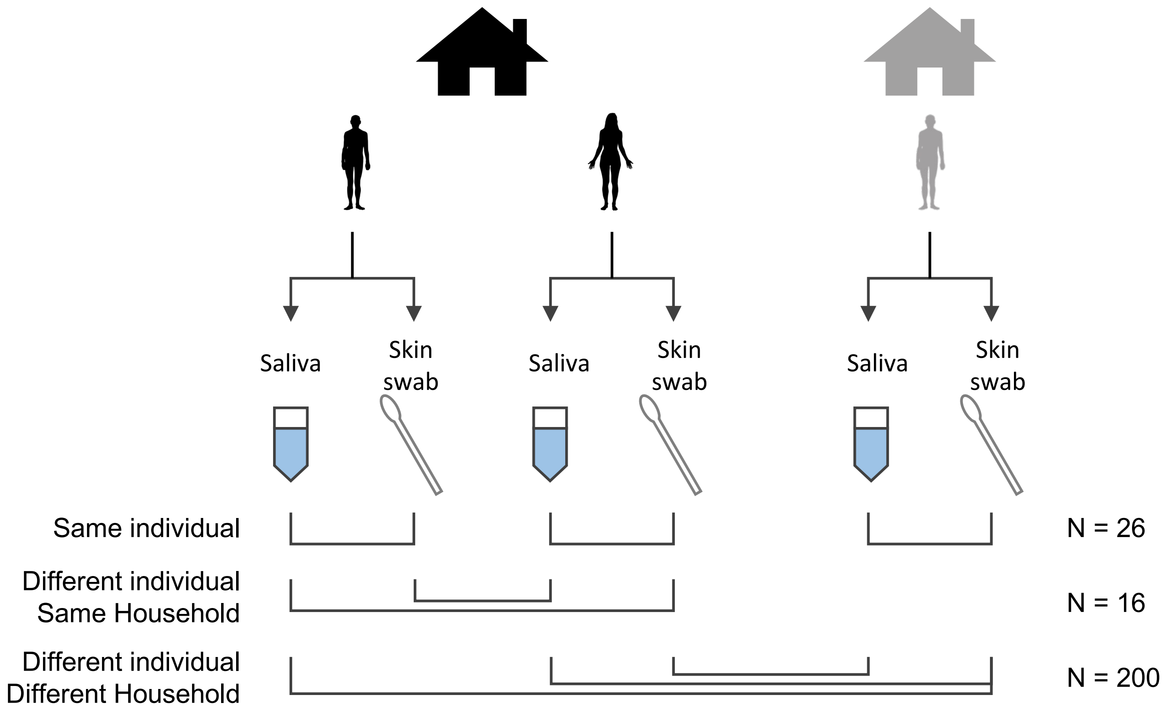

Supplemental Figure S3
Illustration of comparison of the calculation of Bray–Curtis indices. The comparisons were only made between saliva samples and swab samples. N indicates the number of comparisons for the group.

A

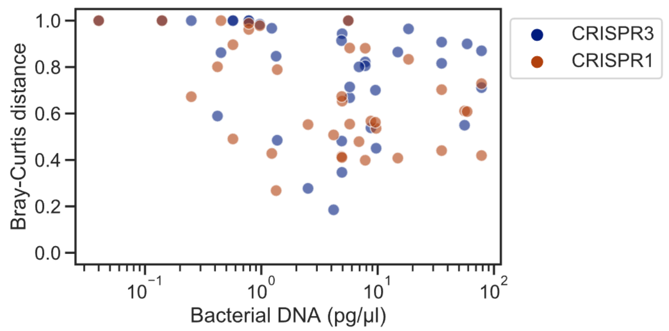

B

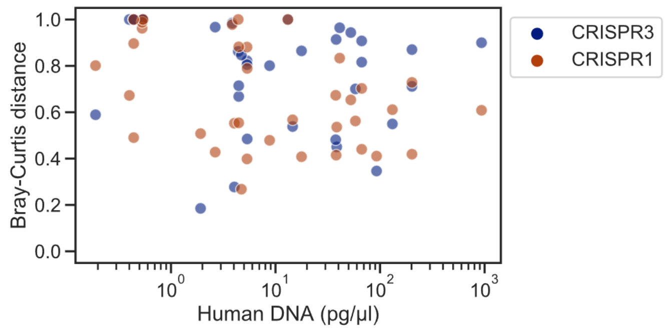

Supplemental Figure S4

(A) Bacterial DNA concentration or (B) human DNA concentration in a sample plotted against the Bray–Curtis distance between the swab and reference saliva samples. Data points for neat and 1:10 diluted samples are shown.

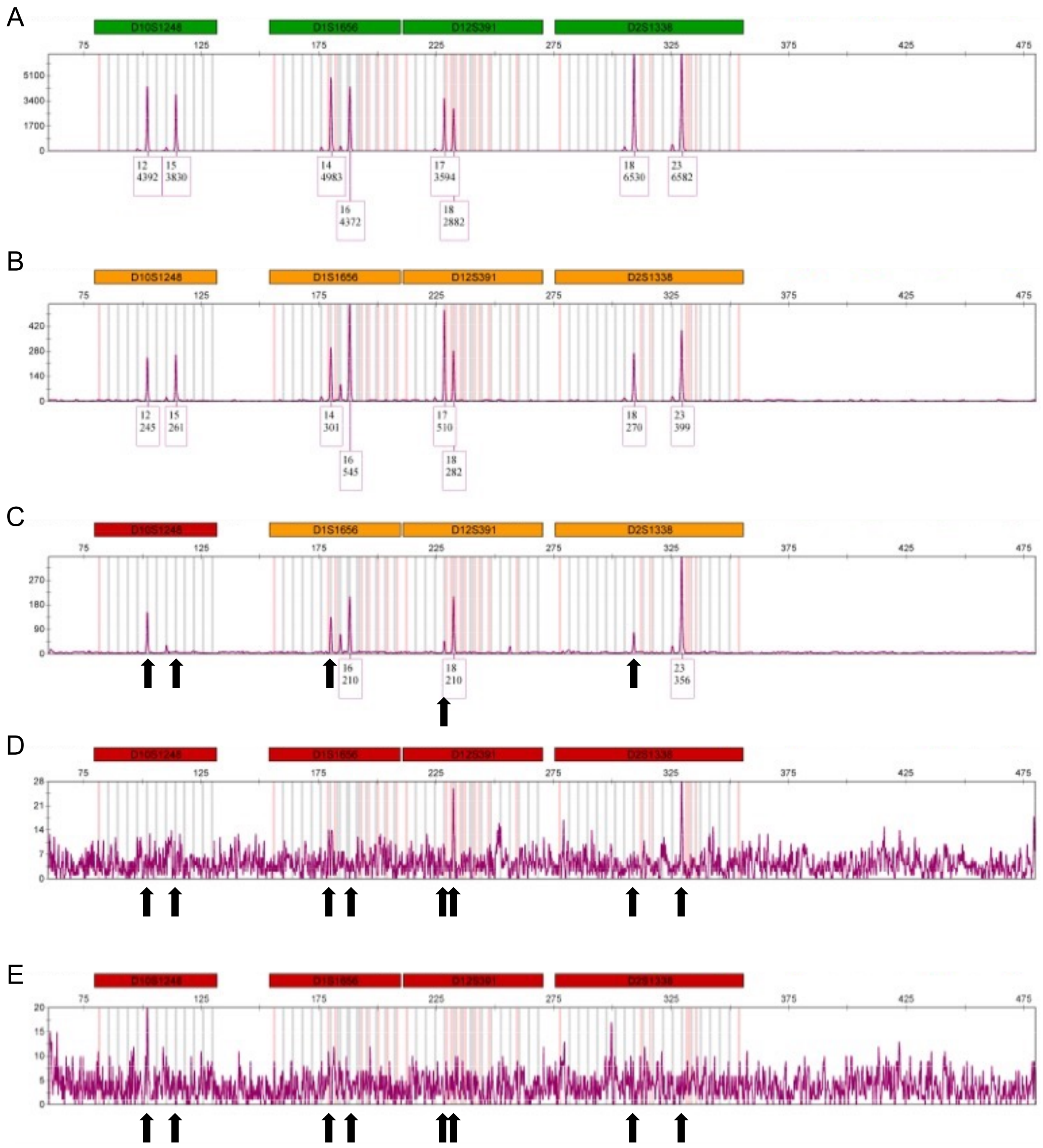

Supplemental Figure S5
Representative profiles of the samples amplified using the GlobalFiler kit and electrophoresed on a 3500xL Genetic Analyzer. The y-axis was normalized to the peak with the highest RFU. Only the results for loci labeled with SID dye are indicated. (A) Saliva, (B) neat skin swab sample taken from the left or (C) right palm, and (D) 1:10 diluted skin swab sample taken from the left or (E) right palm. These samples were derived from the same individual (P03). Arrows indicate the alleles for which the correct alleles were dropped.

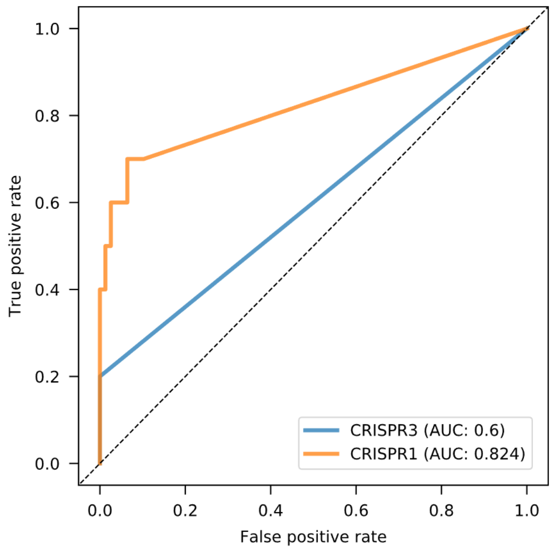

Supplemental Fig. S6

ROC curve analysis demonstrating the performance of metaCRISPR typing for personal identification using diluted samples. Bray–Curtis indices between diluted skin swab samples and saliva samples were used.

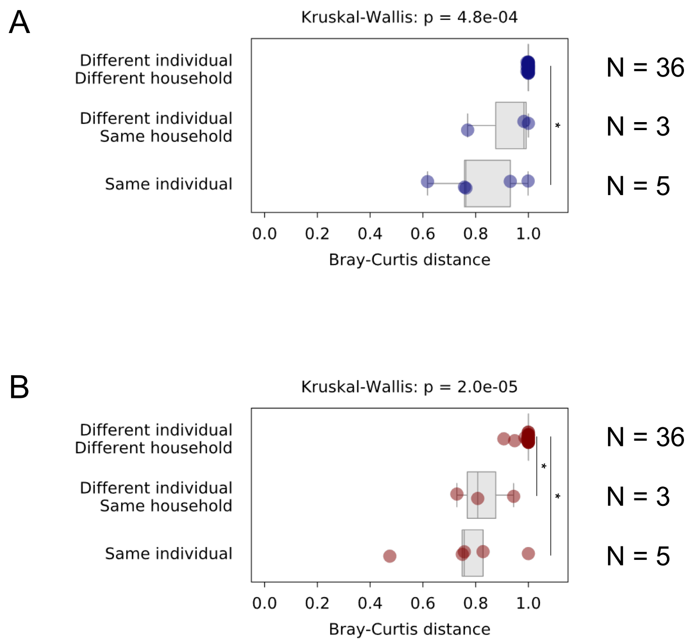

Supplemental Fig. S7

Box plots of the Bray–Curtis dissimilarity indices between/within individuals. The indices were calculated between keyboard samples and saliva samples. The indices were compared using the Kruskal–Wallis test and post-hoc Mann–Whitney U test with Bonferroni correction (*, *adjusted P* < 0.05). (A) CRISPR3 and (B) CRISPR1. N indicates the number of comparisons for the group.
